# Tropical Medicine in China: Bibliometric Analysis Based on Web of Science (2010–2019)

**DOI:** 10.1155/2021/4267230

**Published:** 2021-08-10

**Authors:** Zhen Li, Jingbo Xue, Bin Zheng

**Affiliations:** National Institute of Parasitic Diseases, Chinese Center for Disease Control and Prevention (Chinese Center for Tropical Diseases Research), NHC Key Laboratory of Parasite and Vector Biology, WHO Collaborating Centre for Tropical Diseases, National Center for International Research on Tropical Diseases, Shanghai 200025, China

## Abstract

**Purpose:**

The current study quantitatively assessed research trends in tropical medicine in China via a bibliometric method, which used the Web of Science database to analyse the research-based literature related to tropical medicine published from 2010 to 2019.

**Methods:**

Articles were analysed according to the output and research performance of institutes and countries that collaborated with China. Distribution of keywords was applied to evaluate research trends.

**Results:**

Our findings showed that 3372 articles in the field of tropical medicine have been indexed under the Web of Science database during the past 10 years, indicating that studies pertaining to tropical medicine have been growing from 2010 to 2019. The Chinese Center for Disease Control and Prevention (China CDC) system, which published 549 articles on tropical medicine, may be considered as the flagship of tropical medicine in China. The United States ranked first in internationally collaborative articles with China. Furthermore, Parasite & Vectors, which published 707 papers, emerged as the top journal according to the number of publications.

**Conclusions:**

This study highlights the key institutes and topics pertaining to tropical medicine research in China. Although there has been rapid progress in research on tropical medicine in China, some gaps still remain.

## 1. Introduction

Tropical diseases are prevalent in 149 countries and regions worldwide. Approximately half of the global population is threatened by tropical diseases [1]. The number of infected people is more than a billion, resulting in substantial economic and disease burdens. China, most of which lies in the subtropical zone, is beset by some of the most serious tropical diseases worldwide. Tropical diseases, mainly caused by parasites, have a long history in China and have caused long-term economic losses [2]. In western as well as in underdeveloped areas, tropical diseases often lead to poverty [3, 4]. Imported tropical diseases are also increasing yearly because of the large number of migrant workers arriving in China. The prevention and control of tropical diseases in China, as well as worldwide, are facing severe challenges. In recent years, international health communities have been paying increasing attention to the prevention and control of tropical diseases [5]. Up to now, China has successfully scaled up its efforts to become malaria-free and is certified malaria-free by the WHO [6]. However, China is yet to formulate a systematic, comprehensive policy towards tropical medicine, as a result of which tasks related to research and obtaining international assistance are dispersed among many research institutes and disease control institutions [7]. Organizations that are responsible for researching tropical disease prevention and control rarely collaborate with each other, resulting in a dearth of top-level research publications on tropical disease prevention and control.

Web of Science is a web-based database of citations maintained by the American Institute of Scientific Information. It enables bibliometric analysis of retrieved results. The bibliometric method allows quantitative analyses to be conducted on retrieved documents from multiple perspectives, leading to a better understanding of the status quo of academic research and provides a reference point for exploring a topic in scientific research [8]. A comprehensive understanding of the academic level and influence of a country, institution, or even an individual, as well as the contributions made by these entities to scientific development and academic collaboration, may be determined via an analysis of the number of papers published, the output of documents, and the citations given [9, 10].

The present study was designed to evaluate the current status as well as the potential for the development of the Chinese component of published research in the field of tropical medicine from 2010 to 2019. A bibliometric method was used to analyse tropical medicine trends and performances.

## 2. Methods

### 2.1. Data Sources

The current study, which involved bibliometric research, analysed all articles registered under the subject of “tropical medicine” in the Web of Science between 2010 and 2019. All literature from 2010 to 2019 with “China” in the address field and the words “tropical medicine” was used as a topic within the Web of Science Core Collection database. A total of 3372 records were eventually revealed. This search was performed on June 5^th^, 2020.

### 2.2. Data Analysis

Using bibliometric statistical functions provided by the Web of Science, statistics on the volume, citation, distribution of literature, and distribution of publications, as well as external cooperation between different institutions, were collected. VOS viewer 1.6.11 was used for keyword analysis. ArcGIS 10.1 was used for international collaboration analysis.

## 3. Results

### 3.1. Trend of Articles

**Figure 1:**
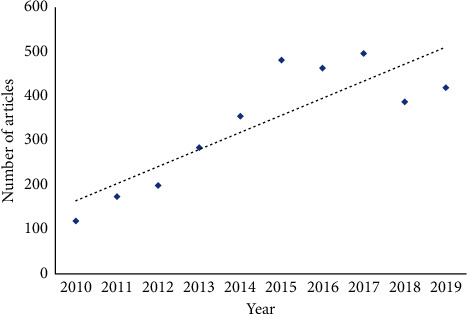
Number of Chinese publications in the period of ten years (2010–2019).

The results of the search strategies used in this study indicated that 3372 records related to Chinese scientists had been indexed under the field of tropical medicine in the Web of Science database over a period of 10 years (2010–2019). The sum of times cited was 32592, while the *h-*index was 59. These findings further indicated that a relative growth had taken place in the number of studies conducted on tropical medicine in China over the past 10 years (Figure 1). The number of studies rose from 119 in 2010 to 419 in 2019, while the number of documents peaked at 495 in 2017.

### 3.2. Document Type of Publication

The distribution of document types, as identified by the Web of Science, was analysed. The 3372 publications produced during the 10-year study period were categorised into nine document types. Articles (2758), comprising 81.79% of total publications, were the most frequently used document type, followed distantly by letters (197; 5.84%), meeting abstracts (187; 5.55%), and reviews (161; 4.77%). Other documents showing less significance were editorials (35; 1.04%), corrections (18; 0.53%), book chapters (13; 0.39%), retractions (2; 0.06%), and new items (1; 0.03%).

### 3.3. International Collaboration

**Figure 2:**
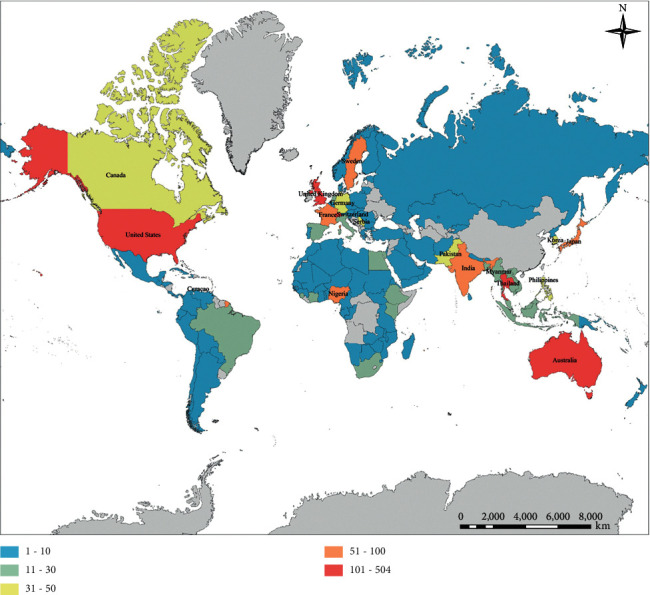
The collaborations of different countries with China. According to the addresses in the article, the number of cooperative publications with China is calculated. Blue represents 1–10 cooperative publications, green represents 11–30 cooperative publications, yellow represents 31–50 cooperative publications, orange represents 51–100 cooperative publications, and red represents 101–504 cooperative publications.

Collaboration with different countries was estimated via the location of affiliation of at least one author of a published paper. Of the 3372 articles with author addresses, 122 were involved in research. A total of 1254 (37.19%) were internationally collaborative articles and 2118 (62.81%) were China-independent articles. Participation of China in publications involving other countries is shown (Figure 2). The top country collaborating with China was USA (504; 14.95%), followed by Thailand (257; 7.63%), UK (186; 5.52%), Australia (165; 4.98%) and Switzerland (106; 3.14%).

### 3.4. Research Institution Comparisons

The contributions of different Chinese institutes were estimated as the affiliation of at least one author. The 3372 articles were published by 2965 institutes that collaborated with 1234 overseas institutes. The top 10 Chinese institutes were ranked by the number of articles, including total publications, sum of times cited, and *h*-index (Table 1). The Chinese Center for Disease Control Prevention (CDC) had the highest number of articles (549), followed by Hainan Medical University (301 articles), Chinese Academy of Agricultural Sciences (CAAS) (225 articles), Fudan University (131 articles), and Sun Yat-sen university (130 articles). Moreover, the *h*-index and sum of times cited of the Chinese Center for Disease Control Prevention ranked the highest. Among the top 10 institutes, 5 were universities and 3 were CDC systems including the Chinese Center for Disease Control Prevention, Shanghai Center for Disease Control Prevention, and Jiangsu Institute of Parasitic Diseases.

In order to analyse international collaboration, Chinese scientists showed a preference towards collaborating with the University of California systems, followed by the Swiss Tropical Public Health Institute and the University of Basel.

### 3.5. Distribution of Publications in Subject Categories and Journals

The 7732 articles were published in 38 journals over 10 years. Parasites & Vectors published the highest number of articles, with 707 accounting for 20.97% of all articles, followed by Asian Pacific Journal of Tropical Medicine (620) and PLOS Neglected Tropical Diseases (386). The top 10 journals accounted for about 90% of all articles, and the impact factors (IF) of journals ranged from 2.5 to 4.5. According to the JCR Category of Tropical Medicine, there were two journals in Quartile 1, two journals in Quartile 2, and one journal in Quartile 3 (Table 2).

### 3.6. The Most Cited Research

An analysis of total citations revealed the top 5 articles and top 5 reviews which were cited the highest number of times in the field of tropical medicine. The article titled “Comparing Diagnostic Accuracy of Kato-Katz, Koga Agar Plate, Ether-Concentration, and FLOTAC for *Schistosoma mansoni* and Soil-Transmitted Helminths” published in the journal PLOS Neglected Tropical Diseases in 2010 is considered by the Web of Science as the most highly cited article (Table 3). This study focused on comparing the diagnostic accuracy of different techniques and sampling efforts, such as single and multiple Kato-Katz thick smears, ether-concentrations, and the FLOTAC method, used to detect and quantify helminth eggs. The performance of the Koga agar plate technique for the detection of helminth larvae was also assessed. Scientists also focused on the surveillance of intestinal parasitic diseases and arthropod-borne diseases. The review titled “A Systematic Review of the Frequency of *Neurocyticercosis* with a Focus on People with Epilepsy” published by PLOS Neglected Tropical Diseases in 2010 is considered by the Web of Science as the highest cited review during the 10 years in question (Table 4).

### 3.7. Research Emphasis and Hotspots

Keyword analysis provides information on research trends as viewed by researchers. The analysis revealed that 6215 words were used in 3372 articles during the ten-year period under study. Certain words, such as China, review, and articles that were considered as not useful for the analysis of research trends, were discarded. Words that appeared less than five times were also removed. Therefore, a total of 270 keywords were used in the final analysis.

The five most frequently used keywords were “malaria,” “*Schistosoma japonicum*,” “*Toxoplasma gondii*,” “prevalence,” and “*tuberculosis*,” indicating that these four tropical diseases have been the focus of research during the past decade.

**Figure 3:**
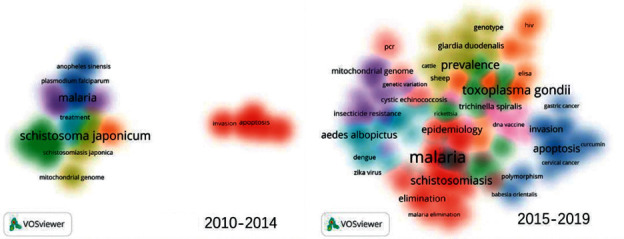
Mapping on cooccurrence of keywords used in Chinese tropical medicine articles. (a) Mapping on cooccurrence of keywords used in Chinese from 2010 to 2014. (b) Mapping on cooccurrence of keywords used in Chinese from 2015 to 2019.

Articles associated with these 270 keywords as well as their distribution were analysed. A change in research topics was noticeable from 2010 to 2019 (Figure 3 ). There were fewer research clusters from 2010 to 2014 than from 2015 to 2019. During the first 5 years, there were seven clusters with different colours. The seven keyword cluster topics were “apoptosis,” “*Toxoplasma gondii*,” “malaria,” “*Schistosoma japonicum*,” “epidemiology,” “*tuberculosis*,” and “vaccine,” respectively. From 2015 to 2019, there were 11 clusters, the topics of which were, namely, “malaria,” “*Haemonchus contortus*,” “apoptosis,” “prevalence,” *“Anopheles sinensis*,” “*Aedes albopictus*,” “*Toxoplasma gondii*,” “*Schistosoma japonicum*,” “*Trichinella spiralis*,” “*Echinococcus granulosus*,” and “Zika virus.”

## 4. Discussion

The Chinese government has increasingly invested in topical medical research over the last decade. In 2003, the Ministry of Education of the People's Republic of China set up the Key Laboratory of Tropical Diseases Prevention and Control at Sun Yat-sen University, making great breakthroughs in the fields of tropical medicine such as dengue fever and malaria. In 2017, the National Research Center for Tropical Diseases, a national research institution specializing in tropical diseases, was set up. Based on the bibliometric analysis of tropical medicine on the Web of Science from 2010 to 2019, the papers published by Chinese researchers are increasing yearly. However, compared to the developed countries, such as the US and UK, the *h*-index and citations remain relatively low. Parasites & Vectors and PLOS Neglected Tropical Diseases are the top two journals in which Chinese researchers have published. Tracing topics and journals with the greatest impact may enable a researcher to find current research topics and improve publication quality. Bibliometric analysis also indicated that the studies conducted by multiple countries and institutes attracted more attention from peers.

In addition to cooperating with developed countries and institutions in Europe and the United States, Chinese scientists also cooperated closely with Asian countries, such as Thailand, India, Japan, Philippines, South Korea, Myanmar, and Indonesia, leading to the formation of the Regional Network for Asian Schistosomiasis and Other Helminth Zoonoses (RNAS^+^) [11]. RNAS, founded in 1998, is funded by the World Health Organization's Tropical Disease Research Program (WHO TDR), the World Health Organization's Center for Neglected Tropical Diseases (WHO NTD), the UK Agency for International Development, and the Canadian International Development Agency, for the purpose of promoting collaboration in research and control strategies for schistosomiasis and other zoonotic helminthiases in Asia. Many countries have incorporated these novel prevention technologies into their own disease control programs. For example, Soares et al. reported that visual technology illustrated the distribution of *Schistosoma japonicum* in the Philippines, enabling the elimination of *S. japonicum* in the Philippines [12]. Leonardo et al. focused on the success of China and Cambodia in eradicating lymphatic filariasis [13].

In the first five years of the past decade, most researchers focused exclusively on a single topic or at most a few topics. This trend has gradually evolved into one which focused on diversified studies during the last five years. A comprehensive analysis of high-frequency words and keyword clustering revealed that “malaria,” “schistosomiasis,” and “toxoplasmosis” were the top three tropical diseases studied.

In 2010, the Action Plan of China Malaria Elimination (2010–2020) (APCME) was officially endorsed by the Ministry of Health in conjunction with 12 other ministries including those of Finance, Education, Science and Technology, Entry-Exit Inspection and Quarantine [14]. The goal of the NMEP is to eliminate local malaria transmission by 2015 (except in some of the Yunnan-Myanmar border areas) and realise malaria elimination across China by 2020. Many surveys of the current malaria epidemic situation have been conducted across Mainland China [15, 16]. Over the past 10 years, Chinese scientists and researchers have developed new, sensitive, and specific malaria detection technologies, such as loop-mediated isothermal amplification (LAMP) and the ultrasensitive reverse transcription-polymerase chain reaction assay [17, 18]. In regard to the development of drug-based treatments, Leong et al. completed the first phase evaluation of the pharmacokinetic/pharmacodynamic interaction of the antimalarial drugs, KAF156, and piperaquine [19]. China's malaria elimination 1–3–7 protocols have been officially entered into the technical documentation of the World Health Organization [20]. Moreover, the Sino-England-Tanzanian malaria on-site prevention and control project has reduced the incidence of malaria as well as associated mortality by 30%, in the communities subjected to intervention [21].

China has conducted schistosomiasis control work for more than 70 years including the work done during the past 10 years. In response to the epidemic nature of schistosomiasis in China, scientists have developed methods to control the source of *S. japonicum* infection, including the innovation of snail control methods, and created methods for detecting and monitoring *S. japonicum* infection [22–26]. In this age of “omics,” omics-based methods have also been employed to develop new diagnostic methods and vaccines, as well as understanding the mechanisms underlying pathogenicity and pathogen-host interactions [27].

Toxoplasmosis is a highly infectious zoonotic parasitic disease. In the past 10 years, researchers have investigated and assessed the risk of *Toxoplasma* infection in animals, such as cats, pigs, civet cats, sika deer, and horses, in different regions [28–32]. In the field of vaccine development, vaccine candidates such as TgDPA, TgROP9, TgMIC3, and TgSAG2 have been found to be most effective [32–34]. In regard to drug development, an investigation of the interaction of the autophagy factor, Atg8–Atg3, in *T. gondii*, demonstrated a mechanism that may induce autophagy in the host, thus showing potential as a target in anti-*Toxoplasma* therapy [35].

An analysis of research topics indicated that “vaccine” was the research hotspot during the first 5 years. For example, M. RCAg-1 developed by Chinese scientists proved to be effective against *T. gondii* [36]. The RTS,S subunit antimalarial drug infection vaccine has entered phase IV clinical trials. Clinical trials have shown that subunit vaccines may exert a partial protective effect on infant clinical and severe malaria [37]. However, parasite vaccines have failed to maintain long-term protection following immunisation, and the safety of some vaccines has been questioned. There has been no breakthrough in vaccine development during the last 5 years, despite several studies being conducted [38]. China still lacks the advanced technology required for vaccine development. Meanwhile, it is expected that the government would provide any necessary resources [39].

Although studies conducted on emerging diseases caused by *H. contortus*, *A. sinensis*, *A. albopictus*, *E. granulosus*, and the Zika virus had been attracting attention from 2015 to 2019, initial studies related to *E. granulosus* began relatively late. However, in 2016, the Chinese government implemented the “National *Echinococcus granulosus* Disease Prevention and Control Plan” (2016–2020), as a result of which funding for *E. granulosus* research has increased. The prevalence of *E. granulosus* in China has been confirmed, and at the molecular level, proteins including Eg-TSP1, EG95, EgM, and TPx have been studied in relation to various aspects associated with new drug targets and vaccine development [34, 40–43].

A few occurrences of dengue fever and Zika virus have been confirmed in China, and monitoring and researching vector-borne diseases such as these are ongoing in many Chinese research institutes. An increasing number of studies have been carried out on vector-borne diseases, and piRNA and miRNA profiles of *A. albopictus*, as well as the mitochondrial genome sequence of *A. sinensis*, have been revealed [44–46]. In addition to the traditional monitoring of spatial distribution of vector-borne diseases, it is also necessary to strengthen the monitoring of vector resistance, with a view to establishing a prediction model using a large data set to further optimise the monitoring mode, resulting in a large volume of theoretical data that may be useful for mosquito vector control [47–49].

Chinese researchers are closely following current topics pertaining to tropical medicine-based research and are focusing on solving urgent issues. Meanwhile, in conjunction with the “Healthy China 2020” and “the Belt and Road” initiatives, China is seeking global collaboration while continuing to invest in medical research. In particular, in the field of tropical medicine, investments made towards developing overseas research centres, such as the China-UK-Tanzania malaria field control project and the China-Australia-Papua New Guinea malaria laboratory project, come to mind. China provides practice sites for research, development, and application of tropical medicine.

However, compared with international research on tropical medicine, there is still a certain gap in China, such as the lack of basic research on tropical medicine, the slow development of diagnostic reagents for related diseases, and the lack of professional personnel for tropical medicine research. These gaps encourage China to increase investment in tropical medicine to reduce the threat of tropical diseases to people's health.

Our article was beset with several limitations. Our search relied only on the Web of Science database and excluded papers published elsewhere, because of which some articles were likely missed due to not being included in the web of Science. Additionally, among the 3372 articles retrieved from the Web of Science Core Collection Database, relevant retrieval was completed on June 5^th^, 2020. Different organizations have different database permissions available, so the retrieved data may be different.

The current study investigated tropical medicine research on the Web of Science database and made several significant determinations related to trends and performances in Chinese tropical medicine research from 2010 to 2019. This study provided a systematic overview of the impact of various topics related to tropical medicine related research projects. A total of 3372 articles were published in 38 journals. The highest number of articles was published in Parasites & Vectors. The United States ranked first among countries that published collaborative articles with China. The CDC system was the flagship of tropical medicine in China, distantly followed by ACCA. In China, research pertaining to tropical medicine is yet in its formative stages. However, some research studies have reached international standards. Keyword analyses indicated that the status of tropical medicine research had evolved from one of single discipline to an interdisciplinary one in China. “*Malaria*,” “S*chistosoma japonicum*,” “*Toxoplasma gondii*,” “prevalence,” and “*schistosomiasis*” were found to be the most popular terms used by Chinese authors. “*Haemonchus contortus,*” “*Anopheles sinensis*,” “*Aedes albopictus*,” “*Echinococcus granulosus*,” and “Zika virus” dominated as new research topics in recent years. The findings of this study may contribute significantly to the advancement of tropical medicine in China.

## Figures and Tables

**Table 1:** The top 15 institutions with tropical medicine related publication and citations, 2010–2019.

Institutions	Total publications	Sum. of times cited	*h*-index
Chinese Center for Disease Control Prevention (CDC)	549	8802	45
Hainan Medical University	301	635	12
Chinese Academy of Agricultural Sciences (CAAS)	225	2952	27
Fudan University	131	981	15
Sun Yat-sen University	130	1625	21
Chinese Academy of Sciences (CAS)	128	1366	21
Shanghai Center for Disease Control Prevention (SCDC)	114	619	13
Jiangsu Institute of Parasitic Diseases (JIPD)	113	1764	25
Southern Medical University China	89	1140	17
Shandong University	87	1161	17
University of California	83	1633	22
Swiss Tropical Public Health Institute	64	1364	21
University of Basel	64	1364	21
Pennsylvania State University	56	741	17
Qimr Berghofer Medical Research Institute	55	1107	18

**Table 2:** The top 10 journals of tropical medicine related publication, 2010–2019.

Rank	Journal	Total publications	5-year impact factor	JCR category in tropical medicine
1	Parasites &Vectors	707	3.342	Q1
2	Asian Pacific Journal of Tropical Medicine	620	1.772	Q3
3	PLOS Neglected Tropical Diseases	386	4.718	Q1
4	American Journal of Tropical Medicine and Hygiene	298	2.655	Q2
5	Infectious Diseases of Poverty	268	3.063	Q1
6	Acta Tropica	251	2.659	Q1
7	Malaria Journal	172	2.882	Q1
8	Tropical Medicine International Health	114	3.057	Q2
9	Southeast Asian Journal of Tropical Medicine and Public Health	86	0.686	Q4
10	Tropical Biomedicine	67	0.778	Q4

JCR = Journal Citation Reports.

**Table 3:** The most cited articles in the field of tropical medicine, 2010–2019.

Title	Authors	Type	Citations	Journal	Year
Comparing Diagnostic Accuracy of Kato-Katz, Koga Agar Plate, Ether-Concentration, and FLOTAC for Schistosoma Mansoni and Soil-Transmitted Helminths	Glinz, Dominik; Silue, Kigbafori D.; Knopp, Stefanie; Lohourignon, Laurent K.; Yao, Kouassi P.; Steinmann, Peter; Rinaldi, Laura; Cringoli, Giuseppe; N'Goran, Eliezer K.; Utzinger, Juerg	Article	134	PLOS Neglected Tropical Diseases	2010
Urbanization Increases Aedes Albopictus Larval Habitats and Accelerates Mosquito Development and Survivorship	Li, Yiji; Kamara, Fatmata; Zhou, Guofa; Puthiyakunnon, Santhosh; Li, Chunyuan; Liu, Yanxia; Zhou, Yanhe; Yao, Lijie; Yan, Guiyun; Chen, Xiao-Guang	Article	132	PLOS Neglected Tropical Diseases	2014
Molecular Surveillance of Cryptosporidium spp., Giardia Duodenalis, and Enterocytozoon Bieneusi by Genotyping and Subtyping Parasites in Wastewater	Li, Na; Xiao, Lihua; Wang, Lin; Zhao, Shuming; Zhao, Xukun; Duan, Liping; Guo, Meijin; Liu, Lili; Feng, Yaoyu	Article	121	PLOS Neglected Tropical Diseases	2012
Concurrent Infections of Giardia Duodenalis, Enterocytozoon Bieneusi, and Clostridium Difficile In Children during a Cryptosporidiosis Outbreak in a Pediatric Hospital in China	Wang, Lin; Xiao, Lihua; Duan, Liping; Ye, Jianbin; Guo, Yaqiong; Guo, Meijin; Liu, Lili; Feng, Yaoyu	Article	118	PLOS Neglected Tropical Diseases	2013
The Neglected Arboviral Infections In Mainland China	Gao, Xiaoyan; Nasci, Roger; Liang, Guodong	Article	117	PLOS Neglected Tropical Diseases	2010

**Table 4:** The most cited reviews in the field of tropical medicine, 2010–2019.

Title	Authors	Type	Citations	Journal	Year
A Systematic Review of the Frequency of Neurocyticercosis with a Focus on People with Epilepsy	Ndimubanzi, Patrick C.; Carabin, Helene; Budke, Christine M.; Nguyen, Hai; Qian, Ying-Jun; Rainwater, Elizabeth; Dickey, Mary; Reynolds, Stefanie; Stoner, Julie A.	Review	213	PLOS Neglected Tropical Diseases	2010
Toxoplasma gondii Infection in Humans in China	Zhou, Peng; Chen, Zhaoguo; Li, Hai-Long; Zheng, Haihong; He, Shenyi; Lin, Rui-Qing; Zhu, Xing-Quan	Review	199	Parasites & Vectors	2011
Soil-Transmitted Helminth Reinfection after Drug Treatment: A Systematic Review and Meta-Analysis	Jia, Tie-Wu; Melville, Sara; Utzinger, Juerg; King, Charles H.; Zhou, Xiao-Nong	Review	171	PLOS Neglected Tropical Diseases	2012
Review: Dengue Fever in Mainland China	Wu, Jin-Ya; Lun, Zhao-Rong; James, Anthony A.; Chen, Xiao-Guang	Review	171	American Journal of Tropical Medicine and Hygiene	2010
Clinical Manifestations Associated with Neurocysticercosis: A Systematic Review	Carabin, Helene; Ndimubanzi, Patrick Cyaga; Budke, Christine M.; Hai Nguyen; Qian, Yingjun; Cowan, Linda Demetry; Stoner, Julie Ann; Rainwater, Elizabeth; Dickey, Mary	Review	150	PLOS Neglected Tropical Diseases	2011

## Data Availability

All data are available upon request to the corresponding author.

## Supplementary Materials

Supplementary Materials *Supplementary Table 1*. The top 10 countries with tropical medicine related publication, *h*-index, and citations, under 2010–2019.
