# Supplementary material for: Tropical Medicine in China: Bibliometric Analysis Based on Web of Science (2010–2019)
Source: J Trop Med. 2021 Aug 10;2021:4267230. doi: 10.1155/2021/4267230 (PMC8371669; doi:10.1155/2021/4267230)
Supplement: Supplementary Materials — Supplementary Table 1. The top 10 countries with tropical medicine related publication, h-index, and citations, under 2010–2019. [file 4267230.f1.docx]

**Supplement Table 1. The top 10 Countries with tropical medicine related publication, *h*-index and citations, 2010-2019**

| **countries** | **Total publications** | ***h*-index** | **the sum of times cited** |
| --- | --- | --- | --- |
| USA | 17959 | 108^(1)^ | 163020 |
| United Kingdom | 7458 | 99 | 94950 |
| Brazil | 7428 | 73 | 70987 |
| India | 4182 | 57 | 30808 |
| China | 3372 | 59 | 32592 |
| Switzerland | 3361 | 81 | 43413 |
| France | 3201 | 76 | 43428 |
| Thailand | 3282 | 60 | 26986 |
| Australia | 2697 | 72 | 34755 |
| Germany | 2330 | 55 | 23241 |

1:The web of science only provides bibliometric analysis of less than 10000 publication. The *H*-index here represents the statistics of published papers from 2010 to 2016 base on 9025 articles.
